# Diverse Ecological Strategies Increase Invasion Resistance in an Experimental Grassland Restoration

**DOI:** 10.1002/ece3.71575

**Published:** 2025-06-11

**Authors:** Adrienne R. Ernst, Daniel J. Larkin, Andrea T. Kramer, Mary‐Claire Glasenhardt, Andrew L. Hipp

**Affiliations:** ^1^ Environmental Science and Studies Berry College Mount Berry Georgia USA; ^2^ Plant Biology and Conservation Northwestern University Evanston Illinois USA; ^3^ Negaunee Institute for Plant Conservation Science and Action Chicago Botanic Garden Glencoe Illinois USA; ^4^ Department of Fisheries, Wildlife, and Conservation Biology University of Minnesota Saint Paul Minnesota USA; ^5^ Nelson Institute of Environmental Studies University of Wisconsin Madison Wisconsin USA; ^6^ Center for Tree Science The Morton Arboretum Lisle Illinois USA

**Keywords:** biotic resistance, diversity‐invasibility, invasion paradox, prairie

## Abstract

Understanding how the characteristics of native plant communities influence invasion is a pressing question, with implications for theory and management. For decades, the primary native community characteristic used in tests of biotic resistance was species richness. However, previous studies have demonstrated that evolutionary history and functional traits shape the invasion process, as ecological theory predicts. Theoretically, restoration projects would benefit from designing seed mixtures around maximizing resistance to invasion. However, there is little empirical evidence on the importance of evolutionary diversity for management and still less guidance for practitioners on effective application of ecological theories. We empirically tested how several native community characteristics (phylogenetic diversity, functional diversity, phylogenetic relatedness, and mean trait values) affected the survival of three introduced invasive species. We explored this question in experimentally restored 15‐species prairie plots with three levels of phylogenetic diversity and two levels of functional diversity. Our experiment also included monocultures of all native species, which were also experimentally invaded. We found evidence that phylogenetic diversity conferred biotic resistance against one invasive species, contributing to reduced biomass in models explaining up to 10% of variance. Tall species better suppressed invaders, with height explaining up to 27% of variation in invader biomass. Surprisingly, we found patterns in leaf and seed traits linked to invasion resistance which were associated with both conservative and resource‐acquisitive strategies. We also found evidence in both the diversity and monoculture plots that invaders were more successful with more closely related native species. Taken together, our results indicate that invasion resistance emerges from nuanced interactions between phylogenetic diversity, functional traits, and community composition, rather than from any single community characteristic. Our results underscore the complexity of biotic resistance and suggest that practitioners should prioritize phylogenetic diversity and strategic species selection when designing restoration plantings to enhance invasion resistance.

## Graphical abstract

This experiment tests the effects of phylogenetic and functional trait attributes on invasion resistance in experimentally restored tallgrass prairie plots. We found that more diverse plots with varied ecological strategies are more likely to suppress invaders.

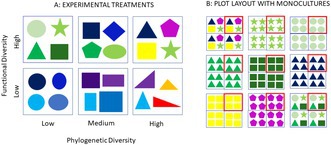

## 1 Introduction

The question of how native species characteristics shape invasion, originally raised in invasion biology's foundational work (Elton 1958), remains an active area in ecology (Peng et al. 2019; Smith and Côté 2019; Stohlgren et al. 2003; Naeem et al. 2000). Elton (1958) observed that invasive species established readily in some biological communities, but not in others–these communities seemed to resist invasion. Understanding what confers this ‘biotic resistance’ to invasive species is crucial from both a management perspective and as a test of ecological theory and how communities assemble. Decades of research in this area have identified noteworthy patterns, particularly with regard to the relationship between species richness and invasion, but these patterns are heavily context dependent and have not yielded sufficient understanding of the factors shaping biotic resistance to allow for predictable and reliable design of native communities to resist invasion.

Biotic resistance has become synonymous with Elton's leading hypothesis, that more diverse native communities are less likely to be invaded, a well studied phenomenon (Beaury et al. 2020; Catford et al. 2009; Richardson and Pyšek 2008; Smith and Côté 2019). Theory suggests that niche space available to the invader drives biotic resistance (i.e., more diverse communities have less niche space available for the invader to exploit) (D'Antonio and Thomsen 2004). However, biotic resistance has primarily been quantified with species richness, an imprecise proxy for the niche (Cadotte et al. 2011). Within community ecology, there has been increasing interest in exploring axes of biodiversity with stronger conceptual links to niche, particularly phylogenetic and functional diversity (Cadotte et al. 2009; Cavender‐Bares et al. 2009; Davies 2021; Gallien and Carboni 2017). Functional diversity quantifies variation in ecologically relevant traits, and higher functional diversity should correlate with greater niche space occupied. Phylogenetic diversity measures shared ancestry among species and, as species relatedness generally reflects ecological difference (Cadotte 2013, though see Mayfield and Levine 2010), may also reflect niche space, potentially including that of unmeasured traits. Both phylogenetic and functional diversity have been shown to correlate with a number of ecosystem services (Thompson et al. 2015); however, there have been few field experiments that have explicitly manipulated phylogenetic and functional diversity in the context of invasion resistance (Byun et al. 2020; Galland et al. 2019). Increasing our understanding of how these axes of diversity influence invasion resistance may simultaneously answer fundamental questions about how species coexist and potentially generate novel strategies for land managers to promote more resilient ecosystems.

An underlying theory related to biotic resistance is that invasion dynamics depend upon the niche overlap between the native community and the invasive species. Elton conceived the notion of empty niches that existed in species‐poor communities and increased their susceptibility to invasion, but the idea can be traced further back to Darwin's work in 1859. He noted that more closely related species were generally more ecologically similar and had similar resource requirements. This led to two opposing conclusions: (1) invasive species are more likely to find a suitable habitat in communities with a closely related resident species; however, (2) ecological similarities would lead to intense competition that would decrease the invasive species' success. Empirical studies have found support for these contrasting expectations, which are encompassed within Darwin's Naturalization Conundrum (Diez et al. 2008; Marx et al. 2016; Pinto‐Ledezma et al. 2020). The finding that phylogenetic distance predicts invasive species establishment underscores that phylogenetic distance generally reflects ecological similarities (though see Cavender‐Bares et al. 2004; Mayfield and Levine 2010), and can be used as a line of evidence in understanding invasion dynamics. Furthermore, testing Darwin's Naturalization Conundrum offers a coarse assessment of the degree to which niche overlap influences invader establishment.

Species coexistence should be governed by a combination of niche‐based processes, like biotic resistance, as well as competitive differences (Mayfield and Levine 2010; Shea and Chesson 2002). If an invasive species possesses strong competitive abilities, it may overwhelm biotic resistance exerted by the native community (Kunstler et al. 2012). Studying traits can give us insight into the competitive landscape and ecological strategies employed by both native and invasive plants. Many plant traits can be placed along a spectrum representing a trade‐off in ecological strategy called the conservation‐acquisition spectrum (Díaz et al. 2016). The conservation end of the spectrum is typified by species that invest resources into longer‐lived structures and defense, while the acquisition end of the spectrum is typified by species that prioritize fast growth and reproduction (Reich 2014; Grime 2006). Many invasive plants fall on the acquisition end of the spectrum with rapid growth and reproduction believed to confer a competitive advantage (Montesinos 2021; Penuelas et al. 2010; Van Kleunen et al. 2010). A common recommendation is to plant native plants with this same strategy because plants with conservative traits will be unlikely to compete with invasive plants employing an acquisitive strategy (Laughlin 2014; Yannelli et al. 2018). However, there has been some evidence to the contrary in grassland ecosystems (Catford et al. 2019; Ernst et al. 2023). Few studies have investigated the degree to which diversity, niche‐based processes, and ecological strategy shape the invasion process.

We investigated how native plant community characteristics affected invasion resistance within an experimental prairie restoration. We established communities with varied phylogenetic and functional diversity, but with species richness held constant. We planted three species of invaders into these plots and tracked their progress for 2 years. In addition to the diversity treatments, we established monocultures of each native species. These monocultures were also experimentally invaded. The monoculture plots enabled us to compare the effect of phylogenetic distances between the native species and invaders, and traits of native species on invader establishment and survival. With these data, we were able to explore how these relationships scaled in the 15‐species diversity treatments and assess the relative importance of biotic resistance, ecological similarity, and competitive differences in the diversity plots.

## 2 Materials and Methods

### 2.1 Experimental Design

**FIGURE 1:**
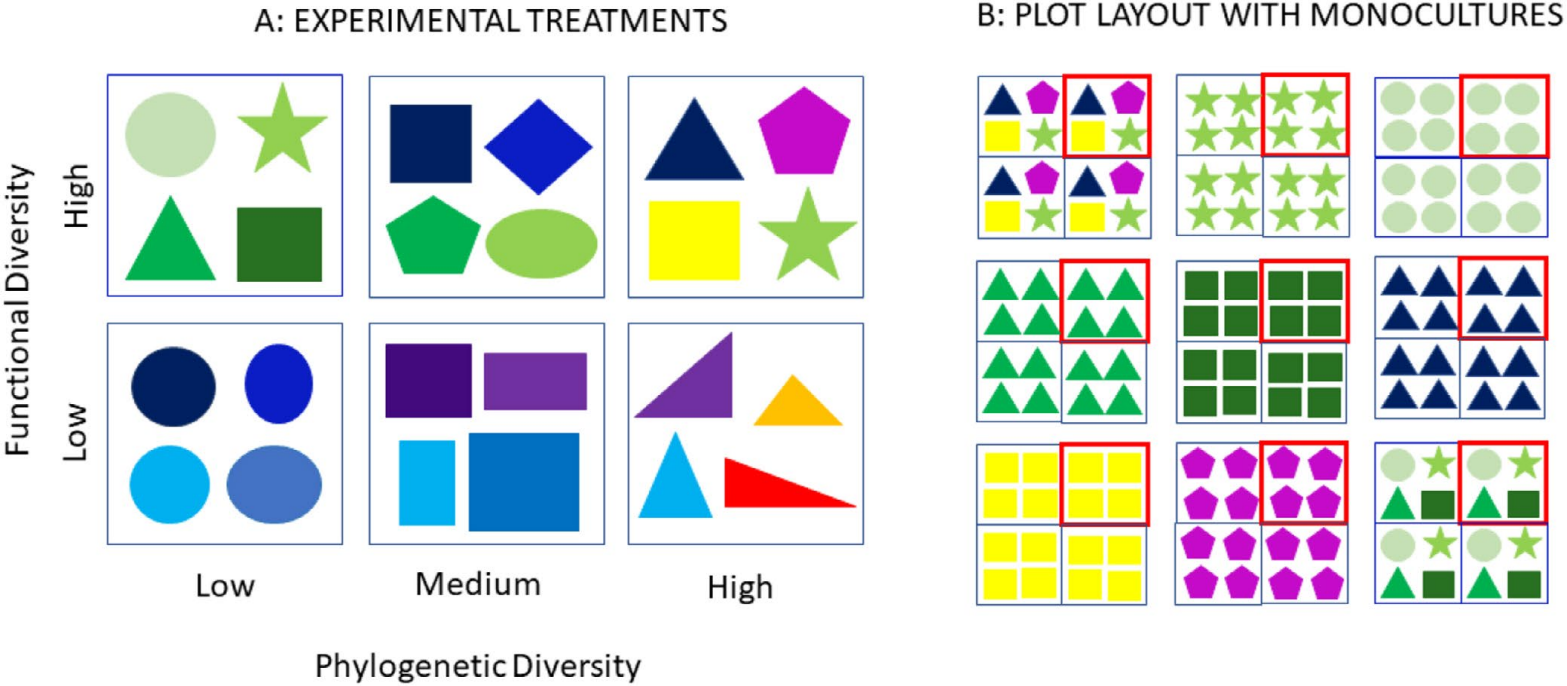
Conceptual figure of experimental design. Species are represented by shape and color. Phylogenetic diversity is represented by colors, species that are more similar in color are more closely related. Functional diversity is represented by shape, species that are more similar in their shape are more functionally similar. (A) We had 15 species communities, depicted as four species communities for visual clarity. For each of the six diversity treatments, we had six different community compositions with each specific community replicated twice. (B) Each species in the diversity treatments was established as a monoculture, with two monoculture plots of each species in the experiment. We had a total of 127 species in the experiment and 254 monoculture plots. We had 72 diversity plots with 36 distinct community compositions with 6 diversity treatments. Each 15 species community was replicated in each quarter of the 4 m^2^ plots. The invasion experiment happened in the northeast corner of each plot, represented by the red square in the figure.

Our experiment was established at The Morton Arboretum in Lisle, Illinois, USA. The full experimental design is described in detail in Hipp et al. (2018) and Karimi et al. (2022). We established 15‐species communities in 2‐m × 2‐m plots with three levels of phylogenetic diversity and two levels of functional diversity (Figure 1). For each diversity level, there were six distinct 15‐species assemblages that were randomly selected. Each community was replicated twice in the experiment for a total of 72 diversity plots (three levels of phylogenetic diversity × two levels of functional diversity × six communities × two replicates). A total of 127 commercially available prairie species used in restoration were included in these diversity plots. Each of the species that occurred in the diversity treatments was also established in twice‐replicated monoculture plots. Soil A‐horizon depth was measured prior to planting and was used to delineate two superblocks, each containing three blocks.

The plots included in this study were all established from plugs in fall 2016. The experiment also included 72 diversity plots established from seed, which were not included in this study. Each plug plot was planted with 60 plugs. In the diversity treatments, the spatial arrangement of the species was randomized but constrained so that each 1‐m × 1‐m quarter of a plot contained the full 15 species. Our invasion experiment took place in the northeast quarter of each plot such that our experimental invaders had the potential to interact with the full community. The plots were weeded of all plants except the native species and invaders that were intentionally planted into each plot.

We selected three species of invaders and planted three individuals from each species into each diversity and monoculture plot in spring 2019. Seed for each species was collected from areas surrounding the experimental site in summer 2018. Seedlings were grown in cone‐tainers in a greenhouse and then hardened off in a hoop house for 2 weeks. Each of the species selected was a biennial and the intent was to collect aboveground biomass of all invaders in summer 2020, although this was complicated by unforeseen circumstances detailed below.

### 2.2 Invader Species

For our experiment, we selected three species across an “invader continuum.” The definition of invasive species varies from nonnative to ecologically harmful to simply undesirable. For our experiment, we chose *Oenothera biennis* , a native species that can be aggressive enough in prairie restorations to warrant control (Havercamp and Whitney 1983); *Daucus carota* , a nonnative species that can be undesirable but is usually not ecologically aggressive in regional restorations; and *Rumex crispus* , a nonnative species that can become aggressive in grassland settings and is a widespread invasive species globally (Cavers and Harper 1964; Zaller 2004).

### 2.3 Data Measurement and Accounting for Differential Invader Responses

All three invaders are considered biennials and were expected to form a basal rosette in their first year and then bolt and bloom in their second year. However, we encountered unexpected challenges with each species (Table 1). A majority of *Oenothera biennis* bloomed in the first field season and, due to time constraints, we were only able to record the number of seed capsules produced by each individual as a measure of lifetime fitness. *Oenothera biennis* plants that survived and bloomed in the second field season had aboveground biomass measured as originally planned. Additionally, many *Rumex crispus* individuals did not bolt in their second growing season, so biomass was collected for the basal rosettes, and biomass analyses were separated by growth form. Finally, *Daucus carota* individuals underwent heavy herbivory by voles at the site, causing extremely low survivorship, particularly in the diversity plots. Only 6 individuals out of 216 planted into the diversity plots survived, so we were ultimately unable to measure the effect of diversity on *Daucus carota* survival or productivity.

**TABLE 1:** Summary of complications encountered for each species, responses studied for each species, and the effect of predictors (positive or negative) on each response.

Species	Unexpected complication	Responses	PD		Seed mass		Height		LCC		LNC		SLA		Phy distance	
			Div	Mono	Div	Mono	Div	Mono	Div	Mono	Div	Mono	Div	Mono	Div	Mono
*O. biennis*	Majority flowered in year 1, only capsule count collected in year 1	Capsule count (year one), survival, biomass (year one and two)			surv (−)	cap (+)	bio (−)	surv (−), cap (−)		cap (+)		bio (+)				
*D. carota*	Most plants were killed by vole herbivory, too few survived in the diversity plots for analysis	Survival and biomass	NA		N	bio (+)				surv (+)	NA	surv (+)	NA		NA	surv (−)
*R. crispus*	Many plants did not flower, biomass had to be separated by growth form (flowered vs. basal)	Survival, basal biomass (monoculture and diversity), flowering biomass (monoculture only)	ba bio (−)		surv (−)			surv (−), fl bio (−)		fl bio (+)		surv (+)	ba bio (+)		surv (−)	surv (−)

*Note:* “PD” stands for phylogenetic diversity. For the responses, “surv” indicates an effect on survival, “cap” indicates an effect on capsule production, “bio” indicates an effect on biomass, “fl” indicates an effect on flowering individuals while “ba” indicates an effect on basal individuals.

### 2.4 Functional Trait Measurement

Functional diversity was measured using 12 continuous traits, 6 categorical traits, 8 binary root traits, seed mass, a habit moisture trait, and genome size. We used the functional trait matrix described in Hipp et al. (2018) as a starting point. That dataset was compiled from published sources (Amatangelo et al. 2014; Sonnier et al. 2014) and supplemented with data gathered for this experiment from nearby prairies (TRY dataset ID 671; Kattge et al. 2020). This original species × traits matrix lacked data for 10.9% of the cells. Unmeasured trait data were imputed using multivariate imputation based on chain equations (MICE) using the mice package v. 3.8.0 (van Buuren and Groothuis‐Oudshoorn 2011).

We attempted to gather specific leaf area (SLA), vegetative height, seed mass, leaf carbon content (LCC), and leaf nitrogen content (LNC) data for all native species that were missing trait data for this suite of traits, although some species had too few surviving individuals to enable trait collection. We used these data to calculate mean trait value for the native species. We also collected data on these traits for the three invader species. SLA, vegetative height, LCC and LNC were all collected from our experiment following the protocols of Pérez‐Harguindeguy et al. (2013). For leaf traits, we sampled 5 leaves from 5 individuals when possible. Height data were collected from at least 10 individuals. LCC and LNC were calculated via combustion with an Elemental Combustion System 4010 (Costech Analytical Technologies, Valencia, CA, USA). Seed mass data were gathered from the Chicago Botanic Garden seed bank database (“Science Collections Database,” 2020).

We implemented models using both the raw trait matrix, with missing values, and the imputed trait matrix. The results were similar for functional diversity; however, we observed differences between the imputed and raw mean trait values sufficient to alter inferences. We present results using only the raw trait data.

### 2.5 Native Species Attributes

Phylogenetic diversity was measured as Faith's PD of the planted community—the summed branch lengths of all species in the community, and the same metric used to design the experiment. Faith's PD measures phylogenetic richness, or the amount of evolutionary history represented in an assemblage (Tucker et al. 2017). PD was calculated using the *picante* package (Kembel 2010). Functional diversity of the planted community was measured via functional dispersion (FDis), calculated using the FD package (Laliberte et al. 2014). FDis is the average distance of species to the community's centroid in PCoA space (Laliberte and Legendre 2010).

We also measured phylogenetic distance between native species and invaders. Phylogenetic distances were calculated using the cophenetic function in the *picante* package (Kembel 2010). For diversity treatments, we calculated the phylogenetic distance of the community to each invader in two ways: Mean Nearest Taxon Distance (MNTD), or the distance between the invader and the most closely related native species, and Mean Pairwise Distance (MPD), the average of the distances between each native species and the invader (Webb et al. 2002).

### 2.6 Linear Mixed Models

We implemented mixed effect models to test how native species' attributes affected invaders' fates following Zuur et al. (2009). We first tested each native species attribute individually. For predictors that had a significant relationship with the response, we then implemented both forward and backward selection using likelihood ratio tests. For diversity treatments, we considered the following predictors: PD, FDis, MPD.i, MNTD.i, SLA, height, seed mass, LCC, and LNC. For monoculture plots, we considered phylogenetic distance and the same five mean trait values considered in the diversity plots. We included block as a random effect.

Due to the differential performance of each invader, the response measures differed between invader models. For all species, survival and aboveground biomass in the second growing season were used as responses. Due to differences in growth form, *Rumex crispus* was split into basal biomass and bolted biomass—only nine individuals flowered in the diversity treatments, so we did not include bolted biomass in the diversity models for *Rumex crispus* . *Oenothera biennis* had an additional response considered, the number of capsules produced by flowering individuals in the first growing season. Because only six individuals of *Daucus carota* survived in the diversity plots, we did not analyze the effect of diversity on *Daucus carota* survival or biomass.

### 2.7 Aster Models

As a way to integrate across the different demographic stages of the three invaders, we also implemented Aster models (Geyer et al. 2007). Aster models enable joint analysis of survival, reproduction, and fitness over a time series. Aster models combine life history components with different probability distributions and take into account the interdependence of different life history stages. We implemented Aster models using the *aster* package in R v.1.0–3 (Geyer 2019).

**FIGURE 2:**
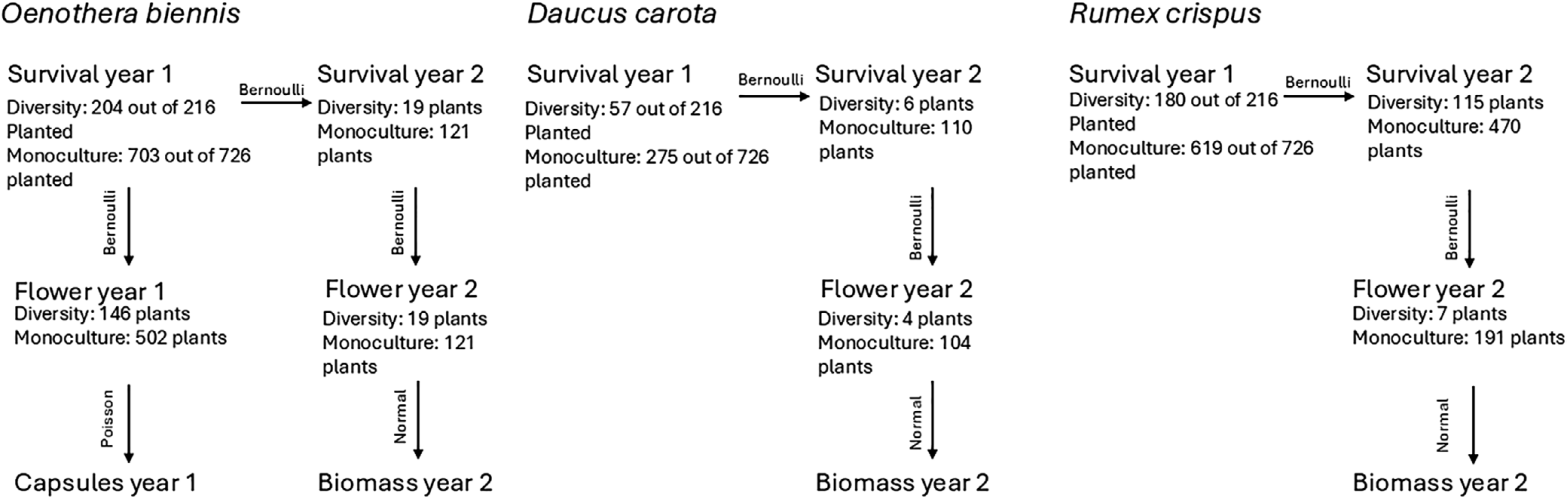
Graphical depiction of Aster model schemes. Arrows are labeled with the distribution used, either Bernoulli, Poisson, or normal distribution. Beneath each life stage is the number of plants that survived or flowered.

The Aster model approach allowed us to test the effects of native species characteristics on each invader's fitness overall, rather than having a set of linear models for each life stage. However, it was developed with fitness and reproduction in mind, which presented some challenges with our dataset. For example, it was not possible to use biomass of both basal and bolted individuals of *Rumex crispus* within the Aster models, as continuous response variables must be assigned to the final node in the life‐history sequence—in our case, flowering. While it would have been possible to structure the model with survival as the final node (thus using basal biomass), doing so would have excluded individuals that bolted, since biomass cannot be meaningfully compared across life‐history stages. We opted to retain bolted individuals in the analysis to better represent the full demographic trajectory of the population, even though this limited our ability to model *Rumex crispus* in the diversity treatments, where so few individuals bolted. As a result, we estimated four total Aster models: *Oenothera biennis* in the diversity plots, *Oenothera biennis* in the monoculture plots, *Daucus carota* in the monoculture plots, and *Rumex crispus* in the monoculture plots (Figure 2).

## 3 Results

### 3.1 Fitness Components Models (Linear Mixed Models)

#### 3.1.1 Native Invader ( *Oenothera biennis* )—Diversity Plots

**FIGURE 3:**
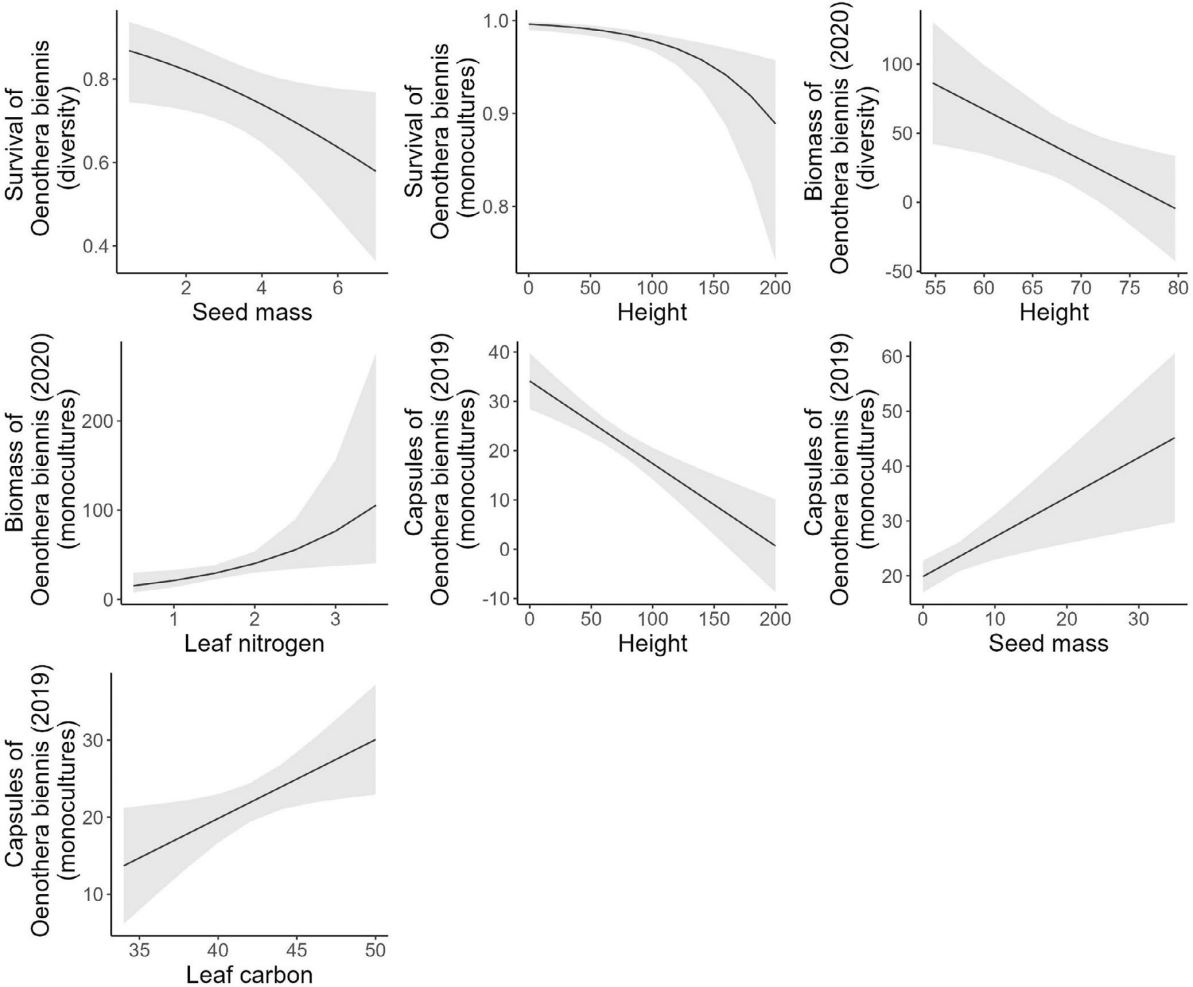
Partial effect plots for all predictors included in the final models for *Oenothera biennis* . Each plot represents the predicted partial effect of the predictor when all other predictors are held constant. The lines indicate the mean predicted by the GLMM and the ribbons indicate the 95% confidence intervals.

In diversity plots, individuals of *Oenothera biennis* were less likely to survive in native communities with heavy seeds (*χ* ^2^ = 4.4, *n* = 216, *p* = 0.03) (Figure 3). None of the measured native community attributes affected the number of capsules produced. *Oenothera biennis* biomass was generally lower in plots with tall native communities (*χ* ^2^ = 5.6, *n* = 19, *p* = 0.02) (Table 2).

**TABLE 2:** Summary of linear mixed models for each invader. Marginal *R* ^2^ indicates the amount of variation explained by the fixed effects (rather than fixed effects + block).

Species	Plot type	Response	Final model	Marginal *R* ^2^	Fixed effect	Standardized coefficient	Standard error
*Oenothera biennis*	Diversity	Survival	Seed mass + (1|block)	0.033	Seed mass	−0.34	0.16
		Capsules	Null				
		Biomass	Height + (1|block)	0.266	Height	−0.51	0.2
	Monoculture	Survival	Height + (1|block)	0.014	Height	−0.22	0.09
		Capsules	LCC + height + seed mass + (1|block)	0.062	LCC	0.1	0.04
					Height	−0.21	0.04
					Seed mass	0.13	0.04
		Biomass	LNC + (1|block)	0.049	LNC	0.22	0.09
*Rumex crispus*	Diversity	Survival	MPD + seed mass + (1|block)	0.07	MPD	−0.37	0.14
					Seed mass	−0.35	0.15
		Biomass (basal)	SLA + PD + (1|block)	0.098	SLA	0.22	0.09
					PD	−0.2	0.09
	Monoculture	Survival	Phy dist + height + LNC + (1|block)	0.052	Phy dist	−0.21	0.08
					Height	−0.27	0.08
					LNC	0.27	0.08
		Biomass (basal)	Null				
		Biomass (bolted)	Height + LCC + (1|block)	0.061	Height	−0.07	0.03
					LCC	0.09	0.03
*Daucus carota*	Monoculture	Survival	LCC + LNC+ phy dist + (1|block)	0.083	LCC	0.46	0.08
					LNC	0.17	0.08
					Phy dist	−0.25	0.08
		Biomass	Seed mass + (1|block)	0.076	Seed mass	0.27	0.1

#### 3.1.2 Native Invader ( *Oenothera biennis* )—Monoculture Plots

In monoculture plots, the likelihood of *Oenothera biennis* survival decreased when it occurred with tall native species (*χ* ^2^ = 6.4, *n* = 762, *p* = 0.01). The number of capsules was lower when *Oenothera biennis* was planted into monoculture plots with species that were tall (*χ* ^2^ = 20.9, *n* = 502, *p* < 0.001), had light seeds (*χ* ^2^ = 8.8, *n* = 502, *p* = 0.003), or contained low leaf carbon (*χ* ^2^ = 5.4, *n* = 502, *p* = 0.02). Biomass of *Oenothera biennis* tended to decrease when it occurred with native species with low leaf nitrogen (*χ* ^2^ = 5.8, *n* = 121, *p* = 0.02).

#### 3.1.3 Nonnative, Aggressive Invader ( *Rumex crispus* )—Diversity Plots

**FIGURE 4:**
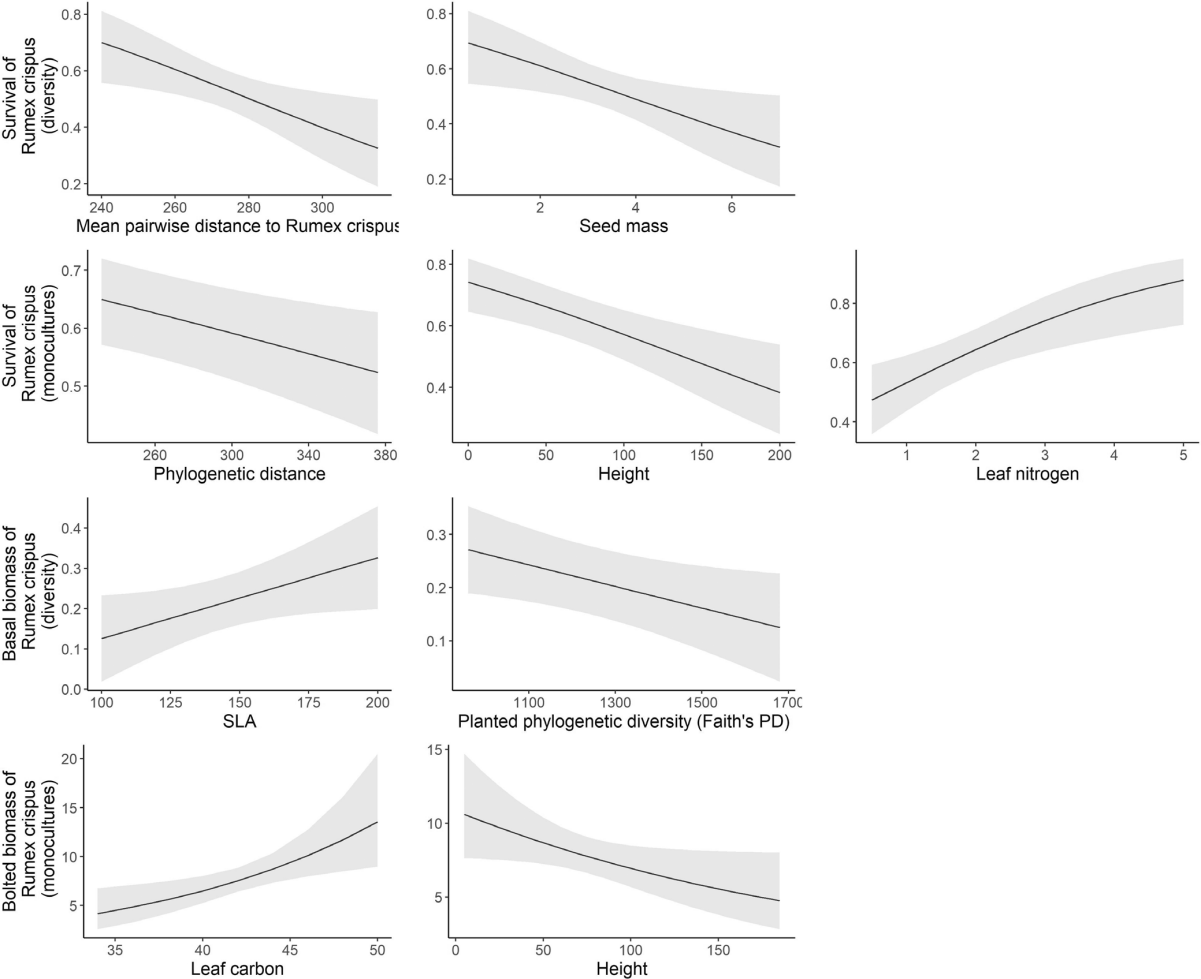
Partial effect plots for all predictors included in the final models for *Rumex crispus* .

Survival of *Rumex crispus* was lower when the native community had heavy seeds (*χ* ^2^ = 6.0, *n* = 216, *p* = 0.01) and was composed of species that were on average more distantly related to *R. crispus* (*χ* ^2^ = 6.8, *n* = 216, *p* = 0.01) (Figure 4). Basal *Rumex crispus* individuals were smaller on average in more phylogenetically diverse communities (*χ* ^2^ = 5.2, *n* = 108, *p* = 0.02) and communities that were composed of species that had low SLA (*χ* ^2^ = 5.8, *n* = 108, *p* = 0.02).

#### 3.1.4 Nonnative, Aggressive Invader ( *Rumex crispus* )—Monoculture Plots

*Rumex crispus* survival decreased when the native monoculture was more distantly related (*χ* ^2^ = 7.0, *n* = 762, *p* = 0.01), tall (*χ* ^2^ = 12.9, *n* = 762, *p* < 0.001), or had low leaf nitrogen (*χ* ^2^ = 10.4, *n* = 762, *p* = 0.001) (Figure 4). There was no effect of any measured native species attributes on basal individuals of *Rumex crispus* in the monoculture plots. Bolted biomass decreased with tall species (*χ* ^2^ = 4.0, *n* = 191, *p* = 0.04) or those with low leaf carbon content (*χ* ^2^ = 7.3, *n* = 191, *p* = 0.01).

#### 3.1.5 Nonnative, Nonaggressive Invader ( *Daucus carota* )—Monoculture Plots

**FIGURE 5:**
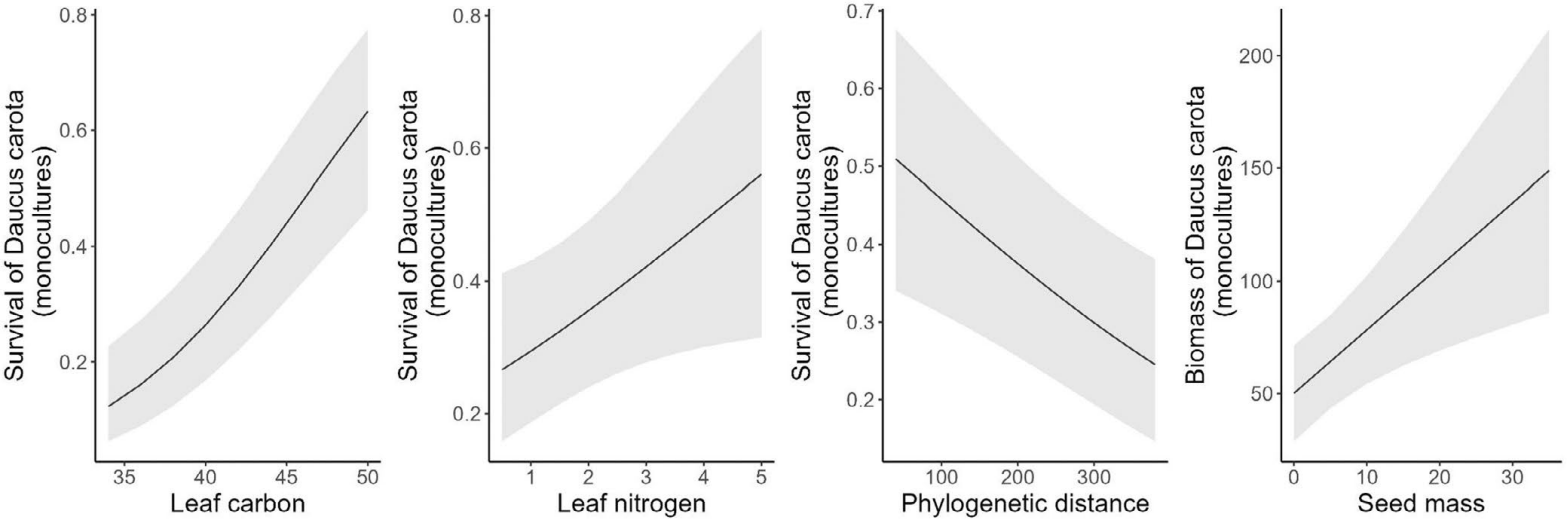
Partial effect plots for all predictors included in the final models for *Daucus carota* .

Likelihood of *Daucus carota* survival decreased when the native species had low leaf carbon (*χ* ^2^ = 31.0, *n* = 762, *p* < 0.001), had low leaf nitrogen (*χ* ^2^ = 4.2, *n* = 762, *p* = 0.04), or was more distantly related (*χ* ^2^ = 9.8, *n* = 762, *p* = 0.002) (Figure 5). Biomass of *Daucus carota* was lower in native monocultures with light seeds (*χ* ^2^ = 8.3, *n* = 104, *p* = 0.003).

### 3.2 Overall Fitness Models (Aster Models)

#### 3.2.1 Native Invader ( *Oenothera biennis* )—Diversity and Monoculture Plots

**FIGURE 6:**
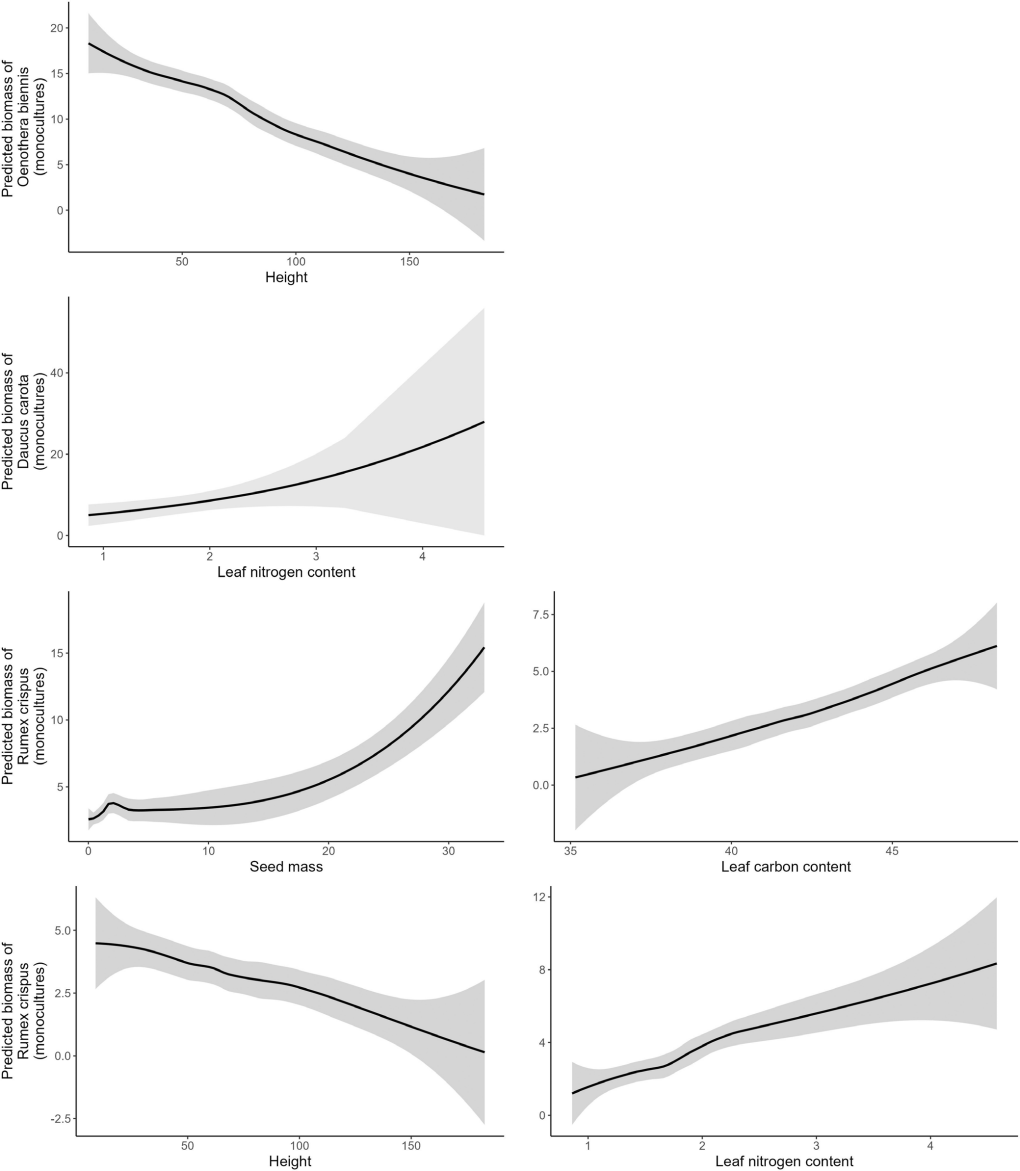
Predicted partial effects of native functional traits on biomass of *Oenothera biennis, Daucus carota*, and *Rumex crispus* from the Aster models.

We found no evidence that any measured native community attributes significantly affected *Oenothera biennis* in the diversity plots. In the monoculture plots, *Oenothera biennis* fitness was lower when it occurred with tall native species (*χ* ^2^ = 6.9, *p* = 0.01) (Figure 6).

#### 3.2.2 Nonnative, Aggressive Invader ( *Rumex crispus* )—Monoculture Plots

We only implemented Aster models for *Rumex crispus* in the monoculture plots. We found that *Rumex crispus* fitness was lower in plots where the native species were tall (*χ* ^2^ = 6.8, *p* = 0.01), had light seeds (*χ* ^2^ = 6.5, *p* = 0.01), or had low carbon content (*χ* ^2^ = 13.3, *p* < 0.001) (Figure 6).

#### 3.2.3 Nonnative, Nonaggressive Invader ( *Daucus carota* )—Monoculture Plots

We found that native species with light seeds (*χ* ^2^ = 4.94, *p* = 0.02) and low carbon (*χ* ^2^ = 9.0, *p* < 0.001) content decreased *Daucus carota* fitness (Figure 6).

## 4 Discussion

Despite the heterogeneous responses of our three invader species, we found some generalizable trends in native species traits and invader performance. First, we found evidence that phylogenetic distance to native species, phylogenetic diversity, and mean trait values of native plant communities all influenced the invasion process in at least one species. We also found that more phylogenetically diverse communities decreased the biomass of one invader species. However, we found evidence that more closely related species may facilitate one another: the survival of two invaders decreased among distant relatives. For one species, we saw that this trend scaled to the diversity treatments: communities that were more distantly related to the invader, on average, decreased the likelihood of invasive species survival. Finally, we saw that mean trait values affected invasion across all invaders, but which traits were most important varied. Invaders generally performed poorly with tall native species or species with low leaf carbon or nitrogen content. We also found that communities with low SLA values reduced *Rumex* biomass. SLA was not linked to invader performance in the other two species. Finally, seed mass had a variable effect on the invasion process: mixed species communities with heavy seeds had higher invader mortality; however, monocultures with light seeds had lower invader biomass. The effects we observed were ecologically meaningful. For example, phylogenetic diversity and SLA together reduced *Rumex crispus* biomass in models that explained up to 10% of the variation, while native species height explained up to 27% of the variation in *Oenothera biennis* biomass. These findings underscore that even modest trait or diversity shifts in plant communities can yield measurable changes in invasion outcomes.

### 4.1 Phylogenetic Diversity Increases Biotic Resistance to a Nonnative Species

We found evidence that increased phylogenetic diversity conferred biotic resistance against *Rumex crispus* . Plots with higher phylogenetic diversity had reduced biomass of basal *Rumex crispus*. This is notable as *Rumex crispus* was our aggressive nonnative invader and the most likely of our study species to be targeted by invasion control methods. Additionally, this is the first field experiment we are aware of that has demonstrated phylogenetic diversity increasing biotic resistance (Galland et al. 2019). Our finding that phylogenetic diversity may confer resistance to invasion in some field contexts has been supported by lab and mesocosm experiments (Feng et al. 2019; Qin et al. 2020) as well as observational studies (Iannone et al. 2016; Yessoufou et al. 2019).

### 4.2 Invaders Survival Increases With More Closely Related Species

Likelihood of invader survival generally increased when the invader was more closely related to the native species. We saw evidence of this for both *Rumex crispus* and *Daucus carota* in the monoculture plots, and for *Rumex crispus* in the diversity plots. In the Darwin's Naturalization Conundrum literature, more closely related invaders are generally thought to succeed because they share adaptations with close relatives that preadapt them to succeed in the same environment (Cadotte et al. 2018; Diez et al. 2008). Rather than reflecting environmental filtering, our experimental design—where we deliberately selected and arranged species—suggests that competitive dynamics may differ with phylogenetic distance. More closely related species may facilitate one another. Previous work has shown that more distantly related species have greater competitive asymmetry, which could promote the coexistence of close relatives (Godoy et al. 2014).

An important caveat to consider is that the three invasive species we studied were from families that were not well represented in our experiment. Among our established native species, we had three species that belonged to the same family as *Daucus carota* (Apiaceae), one species from the same family (and genus) as *Oenothera biennis* (Onagraceae), and no species from the same family as *Rumex crispus* (Polygonaceae). Another relevant consideration is that the tallgrass prairie is characterized by dominance by a small number of families. None of our invaders are from these dominant families. The Asteraceae—the Sunflower Family, which represents approximately ⅓ of the species planted into this experiment—can have pronounced effects on community structure and dominate prairies in early successional stages (Ernst et al. 2023; Hipp et al. 2018; Larkin et al. 2015; Schramm 1990). Many of the Poaceae—the Grass Family, also a tallgrass prairie dominant—had notably lower survival of invaders beneath their thatch in the second year. It may be that phylogenetic distance to the invaders is entangled with phylogenetic identity (family or clade membership) of these dominant prairie families (Larkin et al. 2023).

### 4.3 Height Looms Large in Competitive Landscape

Multiple lines of evidence suggest that tall native species better suppressed the invaders. From our linear mixed models, we saw that communities composed of tall species had lower *Oenothera biennis* biomass. In the monoculture plots, we found that when planted into tall monocultures, *Oenothera biennis* had lower survival, capsule production, and overall fitness, and *Rumex crispus* had lower survival, biomass, and overall fitness. Height has a well‐established trait hierarchy: as taller species shade out shorter species, the difference in height between neighboring species determines how much light each receives (Keddy and Shipley 1989; Kunstler et al. 2012; Fried et al. 2019). Planting taller native species may consequently be one of the most effective strategies to suppress invasive species.

### 4.4 Tortoise and the Hare: More Conservative Leaf Traits May Win out Against Invaders

We found that invaders performed poorly when native species had leaf traits on the resource conservation end of the conservation‐acquisition spectrum. Leaf nitrogen is strongly linked to net CO_2_ assimilation rate (Schulze et al. 1994), likely because most organic leaf nitrogen is used for photosynthetic machinery (Evans and Seemann 1989). SLA is also linked to leaf nitrogen and photosynthetic rate. SLA correlates with the amount of light a leaf can intercept per unit of mass. Leaves with higher SLA tend to have higher photosynthetic capacity, which is in turn linked to leaf nitrogen (Wright et al. 2004). High SLA and high nitrogen are linked to leaves that are cheaper to construct, grow faster, and have a shorter lifespan. In the monoculture plots, native species with low leaf nitrogen had a lower likelihood of survival for both *Daucus carota* and *Rumex crispus* , and *Oenothera biennis* individuals had lower biomass. *Rumex crispus* had lower biomass in communities with low SLA values.

A well‐documented invasion strategy is to prioritize resource acquisition, putting carbon gains toward fast growth that maximizes photosynthetic rate rather than “investing” resources in longer‐lasting leaves or herbivory defense (Montesinos 2021; Penuelas et al. 2010; Van Kleunen et al. 2010). One approach to suppressing invasive species is to plant similarly “fast” native plants that can outcompete invaders (Laughlin 2014; Yannelli et al. 2018). However, our research suggests that more conservative leaf traits confer a competitive advantage. Most invasion studies that have found “fast” species are more competitive have been conducted in highly modified areas with high nutrient availability. These conditions favor fast growth and reproduction rates. Our study and others that have found more conservative traits to be more competitive against invaders have taken place in lower resource environments with a disturbance regime that aims to approximate historical patterns (Catford et al. 2019; Ernst et al. 2022).

### 4.5 Lower Investment in Leaf Carbon Pays Off

We found that monocultures with low leaf carbon content decreased capsule production in *Oenothera biennis* , basal biomass of *Rumex crispus* , and fitness in both *Daucus carota* and *Rumex crispus* . Leaf carbon content is frequently used as a proxy for structural investment in the leaf (Poorter 1994; Poorter et al. 2006). In our study, this would suggest that species that invest less in their leaves and have a shorter leaf lifespan are more competitive against invaders. This contradicts the trends we found in SLA and leaf nitrogen and suggests an alternate strategy wherein “faster” species may also exert competitive pressure against invaders.

The relationship between leaf traits and invasion resistance is complex, and it challenges simplistic management recommendations for one suite of traits or a particular ecological strategy. While a common invasion strategy involves prioritizing rapid resource acquisition and fast growth, our research found evidence that traits associated with a conservative strategy (low SLA and low leaf nitrogen) and traits associated with a resource acquisitive strategy (low leaf carbon) may both confer resistance to invasion. This effect appears context‐dependent, with our results differing from studies conducted in more disturbed and high‐nutrient environments. These results illustrate the complexities in plant community dynamics. Different mechanisms of competition may operate simultaneously through different traits. For example, low SLA and leaf nitrogen may confer competitive advantages through resource use efficiency and persistence in low‐resource conditions, while low leaf carbon might allow for rapid growth and resource preemption during critical establishment phases. Rather than contradicting ecological theory, our finding that both acquisitive and conservative traits confer invasion resistance highlights that multiple ecological strategies can be effective against invaders. Planting species across a range of ecological strategies may maximize invasion resistance more effectively than focusing on a single strategy type.

### 4.6 Dissecting the Role of Seed Mass

Communities with species with heavy seeds had lower invader establishment based on survival of *Rumex crispus* and *Oenothera biennis* . However, monocultures with light seeds had smaller *Daucus carota* individuals, lower capsule production by *Oenothera biennis* , and reduced overall fitness in *Daucus carota* and *Rumex crispus*. While seed mass is linked to competitive differences in early life stages (Grime and Jeffrey 1965; Turnbull et al. 1999), the significance of differences in seed mass in our experiment is likely more reflective of differences in life history strategy associated with seed mass. Our experiment bypassed the germination and emergence bottlenecks—the native species in this experiment had been planted as plugs 3 years prior to the invader introduction, and the invaders themselves were introduced as seedlings. A conservation‐acquisition spectrum has been proposed for seed mass, as species with heavy seeds tend to be larger, live longer, and have lower reproductive output (Moles 2018; Moles and Westoby 2006). The communities of more conservative species—that is, heavier‐ and larger‐seeded species—may be more likely to shade out and competitively exclude invaders altogether. More acquisitive species at the other end of the spectrum are likelier to be annuals or otherwise reproduce quickly, so they may reach higher densities more quickly. The more acquisitive species may be less likely to competitively exclude invasive species but may exert more competitive pressure during establishment that could result in reduced fitness and biomass as we observed in the monoculture plots.

### 4.7 Higher Herbivory of *Daucus carota* in Diverse Communities

We observed high mortality of *Daucus carota* due to vole activity at our field site; however, the mortality was substantially higher in the diversity plots than in the monoculture plots. While this does not reflect an effect of phylogenetic or functional diversity, it is an interesting diversity effect. Diverse communities may attract or maintain higher herbivore populations (Barbosa et al. 2009). While our study was not designed to test herbivory dynamics specifically, this pattern suggests that biotic resistance can operate through multiple mechanisms, not only plant competition but also herbivory.

### 4.8 Challenges Associated With Variable Invader Response

The unexpected responses of our invader species (early flowering in *Oenothera biennis*, delayed bolting in *Rumex crispus* , and high herbivory in *Daucus carota* ) reflect the complexity of invasion dynamics in field settings, but they also created analytical challenges. These varied responses allowed us to observe how invasion processes operate across different life history stages, and we found consistent findings across multiple species and response variables. This consistency despite the varied invader responses suggests that we identified generalizable patterns. However, our study was limited by these challenges. The differential responses lowered our statistical power from the original design. More importantly, we originally designed this study to do more direct comparisons between the monoculture and diversity plots. However, the differences in mortality and life history stage between monoculture and diversity plots precluded us from using the performance of the invaders in the monoculture plots to set up expectations for performance in the diversity plots. Such an analysis would have offered additional insight into the mechanisms of diversity *sensu* Fargione and Tilman (2005). Future studies could explore this question to understand the drivers of invasion resistance, and we would encourage the use of pilot studies to establish that invaders behave similarly in the diversity and monoculture plots.

## 5 Conclusion

Our study demonstrates a complex interplay between native community characteristics and invasion and demonstrates that there are multiple pathways to resisting invasive species. We found evidence that both acquisitive and conservative strategies suppressed invaders. Rather than focusing on introducing fast‐growing, competitive native species that can “beat invaders at their own game,” we suggest that planting species with a diverse suite of ecological strategies may be more likely to increase invasion resistance overall. This is further supported by our finding that more phylogenetically diverse plots better resisted the aggressive nonnative invader in our study. There have been few experimental tests of how phylogenetic diversity affects invasion resistance to date. Our study suggests that managing for a more phylogenetically diverse community may reduce the survival and growth of unwanted species in addition to increasing other key ecosystem functions.

## Author Contributions

**Adrienne R. Ernst:** conceptualization (lead), data curation (lead), formal analysis (lead), funding acquisition (supporting), methodology (lead), software (lead), validation (lead), writing – original draft (lead). **Daniel J. Larkin:** conceptualization (supporting), formal analysis (supporting), funding acquisition (lead), investigation (equal), methodology (supporting), project administration (equal), supervision (supporting), visualization (supporting), writing – original draft (supporting), writing – review and editing (supporting). **Andrea T. Kramer:** conceptualization (supporting), formal analysis (supporting), funding acquisition (supporting), investigation (supporting), methodology (supporting), project administration (supporting), supervision (lead), writing – original draft (supporting), writing – review and editing (lead). **Mary‐Claire Glasenhardt:** conceptualization (supporting), methodology (supporting), project administration (supporting), supervision (supporting), writing – review and editing (supporting). **Andrew L. Hipp:** conceptualization (lead), formal analysis (supporting), funding acquisition (equal), investigation (supporting), methodology (supporting), project administration (equal), supervision (equal), visualization (supporting), writing – original draft (supporting), writing – review and editing (supporting).

## Conflicts of Interest

The authors declare no conflicts of interest.

## Data Availability

Data and metadata are available at Dryad (DOI: https://doi.org/10.5061/dryad.2jm63xt0j).
